# Meta-analysis of the relationship between single nucleotide polymorphism of IL-10-1082G/A and rheumatic heart disease

**DOI:** 10.18632/oncotarget.23901

**Published:** 2018-01-03

**Authors:** Weiran Dai, Ziliang Ye, Haili Lu, Qiang Su, Hui Li, Lang Li

**Affiliations:** ^1^ Department of Cardiology, the First Affiliated Hospital of Guangxi Medical University, Guangxi Cardiovascular Institute, Nanning, Guangxi, China; ^2^ Guangxi Medical University, Nanning, Guangxi, China; ^3^ Department of Infectious Diseases, the First Affiliated Hospital of Guangxi Medical University, Nanning, Guangxi, China

**Keywords:** IL-10-1082G/A, single nucleotide polymorphism, rheumatic heart disease, correlation, meta-analysis

## Abstract

**Background:**

The results showed that there was a certain correlation between the single nucleotide polymorphism of IL-10-1082G/A and rheumatic heart disease, but there was no systematic study to verify this conclusion.

**Aims:**

Systematic review of the association between single nucleotide polymorphism of IL-10-1082G/A locus and rheumatic heart disease.

**Materials and Methods:**

Computer retrieval PubMed, EMbase, Cochrane Library, CBM, CNKI, VIP and Data WanFang, the retrieval time limit from inception to June 2017. A case control study of single nucleotide polymorphisms and rheumatic heart disease in patients with rheumatic heart disease in the IL-10-1082G/A was collected. Two researchers independently screened the literature, extracted data and evaluated the risk of bias in the study, and using RevMan5.3 software for data analysis.

**Results:**

A total of 3 case control studies were included, including 318 patients with rheumatic heart disease and 502 controls. Meta-analysis showed that there was no correlation between IL-10-1082G/A gene polymorphism and rheumatic heart disease [AA+AG VS GG: OR = 0.62, 95% CI (0.28, 1.39), *P* = 0.25; AA VS AG+GG: OR = 0.73, 95% CI (0.54, 1.00), *P* = 0.05; AA VS GG: OR = 0.70, 95% CI(0.47, 1.05), *P* = 0.08; AG VS GG: OR = 0.65, 95% CI (0.22, 1.92), *P* = 0.43; A VS G: OR = 0.87, 95% CI (0.71, 1.06), *P* = 0.17].

**Conclusions:**

When AA is a recessive gene, the single nucleotide polymorphism of IL-10-1082G/A is associated with the presence of rheumatic heart disease. Due to the limitations of the quantity and quality of the included literatures, the further research results were still needed.

## INTRODUCTION

Rheumatic heart disease (RHD) is a class of autoimmune diseases caused by beta hemolytic group A induced by Streptococcus [1–6]. The results of the study show that the streptococcus antigens can simulate the human protein (especially in the heart of the protein) molecules, which lead to RHD patients have their own immune response [7–9]. Cardiac involvement can cause inflammation of the heart, the heart and the heart, resulting in permanent damage to the heart valve and eventually death due to heart failure [10, 11]. Despite the continuous improvement of the modern medical environment, the RHD is still one of the important diseases causing human deaths in underdeveloped areas. According to incomplete statistics, the number of RHD patients around the world is approximately 16000000 [12], and the annual number of new cases and deaths are growing, RHD to become one of the world’s public health problems.

Domestic and foreign research results show that: RHD is a disease caused by genetic [13, 14], environmental [15, 16] and autoimmune factors [17, 18], but the specific mechanism is unclear. Many scholars have carried out some research on RHD at the gene level, among them, the single nucleotide polymorphism of IL-10-1082G/A site is one of the key content of the research. Settin et al. pointed out that: there is a certain correlation between the IL-10-1082G/A site single nucleotide polymorphism and the incidence of RHD [19] [20–22]. But H Chou [23], a Chinese scholar, found that there was no correlation between the IL-10-1082G/A gene polymorphism and the incidence of RHD in the Han population of Taiwan. Because there is a big gap between the results of a single study, and lacking of effective theoretical guidance.

This study used systematic evaluation method to analyze the correlation between IL-10-1082G/A and RHD, and provide some reference for the pathogenesis and genetic susceptibility of RHD from the perspective of evidence-based medicine.

## MATERIALS AND METHODS

### Data and methods

#### Inclusion and exclusion criteria

Inclusion criteria: ① The correlation between IL-10-1082G/A site single nucleotide polymorphism and rheumatic heart disease; ② Study type: case control study; ③ Each study should provide a direct or indirect ratio (OR) value of the two groups of genotype frequencies; ④ Patients with rheumatic heart disease, including mitral valve disease, aortic valve disease and combined valvular disease, patient’s age, sex, combined disease, family history is not limited; In the control group, most of the patients were healthy, and the residents were randomly selected; There was no blood relationship between the subjects in each group; ⑤ The genotype distribution of the control group was consistent with the Hardy-Weinberg (H-W) genetic equilibrium; ⑥ Research object is human.

### Exclusion criteria

① Perspective study; ② Repeated reports; ③ Abstracts, reviews, and information is not all in the form of abstracts; ④ Zoopery; ⑤ Information is incomplete or unable to extract the data from the literature.

### Retrieval strategy

All the subjects were used to retrieve the database. English search terms was rheumatic heart disease, disease, rheumatic heart or diseases, IL-10-1082, mutation, polymorphism, variant, as shown in Table 1. Chinese search term for rheumatic heart disease, gene polymorphism, IL-10-1082. The retrieval time limit from inception to June 2017.

**Table 1:** PubMed retrieval strategy

#1	Rheumatic Heart Disease or Disease, Rheumatic Heart or Diseases, Rheumatic Heart or Heart Disease, Rheumatic or Bouillaud Disease or Disease, Bouillaud
#2	IL 10-1082
#3	mutation
#4	polymorphism
#5	variant
#6	#1 AND #2 AND (#3 OR #4 OR #5)

### Literature screening, data extraction and risk assessment of bias

Two researchers independently screened the literature, extracted data and evaluated the risk of bias in the study. In case of disagreement, discuss the solution or hand in the decision by the third party. Extracting data from the data of self-made data. The main contents including: ① Included in the study of the basic information, including research topics, the first author, published a magazine and time, etc; ② Key elements of risk assessment of bias; ③ Distribution frequency of genotype in case group and control group; The bias risk was evaluated by NOS scale (Scale Newcastle-Ottawa).

### Statistical analysis

Meta-analysis was carried out using RevMan 5.3 software. First of all, the control group was included in the study of the genetic model for the weinberg hardy balance (HWE) test, if *P* < 0.05, then the control group genotype does not meet HWE. Then 5 genetic models were calculated, respectively, including dominant model (AA+AG vs GG), implicit model (AA vs AG+GG), codominant model (AA vs GG), codominant model (AG vs GG) and Allele model (A vs G). The OR value and its 95%CI were used to analyze the effect of combined analysis, and the test level of = 0.05 was analyzed by Meta. The heterogeneity between the results was using the chi square test for analysis (test set the standard for alpha = 0.1), and combined with I^2^ quantitatively determine the size heterogeneity. If the research results were not statistically different, the fixed effect model was used for Meta-analysis. If the research results were statistically different, the random effects model was used to analyze the effects of Meta on the exclusion of significant clinical heterogeneity. Obvious clinical heterogeneity was treated by subgroup analysis, such as the different subgroups of ethnic groups, or sensitivity analysis, and other methods of treatment, or descriptive analysis [24–27].

## RESULTS

### Literature search results

**Figure 1:**
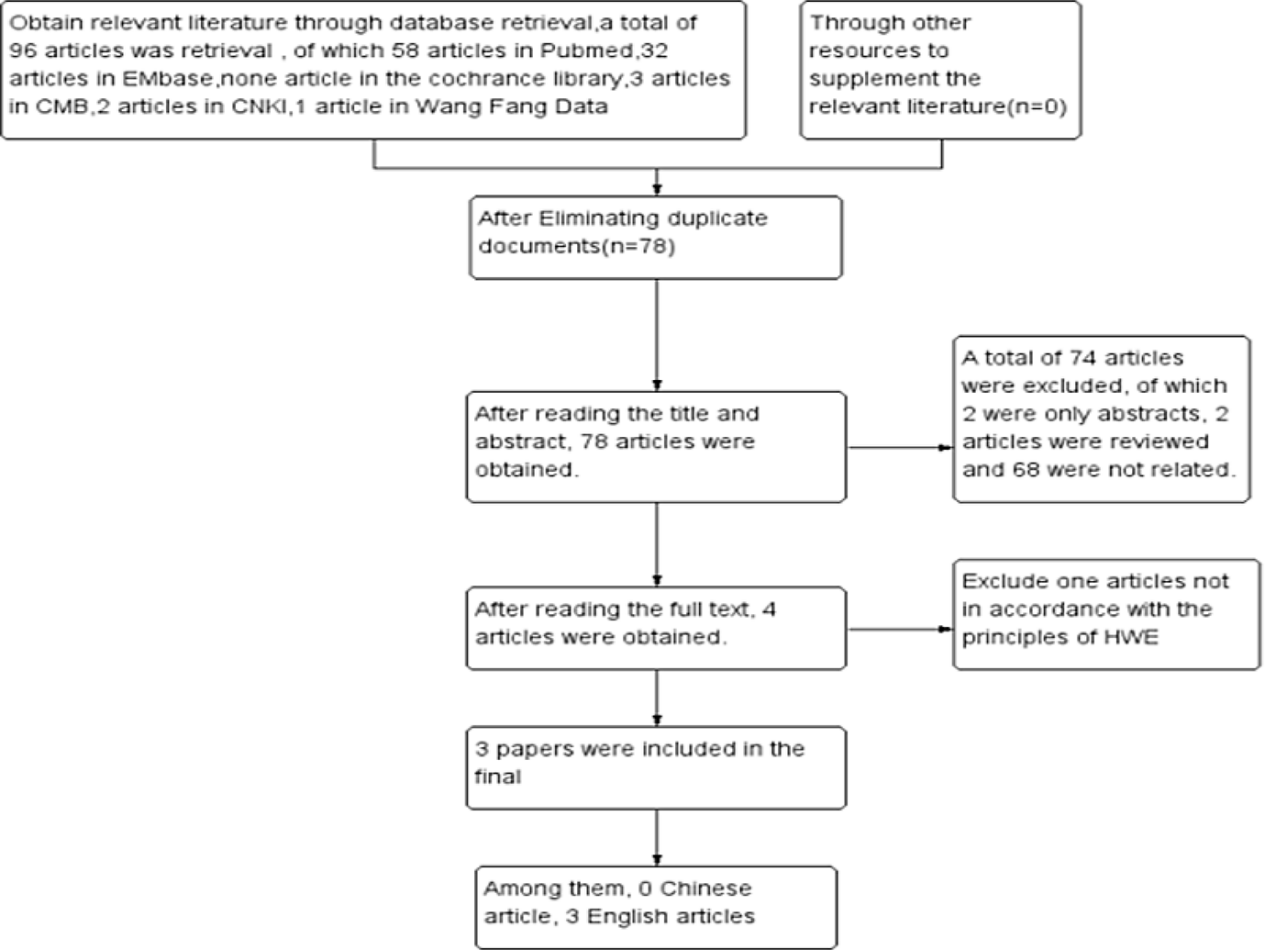
Process and results of literature selection

A total of 96 articles were retrieved, after layer by layer screening, the final 3 case control studies were included [28–30], including three English literatures and 0 Chinese literatures. A total of 318 patients with rheumatic heart disease and 502 control subjects, literature screening process and results are shown in Figure 1.

### The basic characteristics of the included studies and the results of the risk assessment of bias

One article come from Saudi Arabia, the average age of the case group was (19.2 + 5.2) years old, the average age of the control group was (20.6 + 4.5) years old, PCR method was used to detect IL-10-1082G/A, the genes of the control group were accord with Hardy-Weinberg equilibrium, Newcastle-Ottawa Scale score of 9 points; one article come from Egypt, The age of the study population ranged from 5∼18 years old, PCR method was used to detect IL-10-1082G/A, the genes of the control group were accord with Hardy-Weinberg equilibrium, Newcastle-Ottawa Scale score of 8 points; one article come from Pakistan, the average age of the case group was (30 ± 14.5) years old, the average age of the control group was (45 ± 12.7) years old, PCR method was used to detect IL-10-1082G/A, the genes of the control group were accord with Hardy-Weinberg equilibrium, Newcastle-Ottawa Scale score of 9 points. As shown in Table 2.

**Table 2:** The basic characteristics of the included studies and the results of the risk assessment of bias

Inclusion study	Country	Age	Gene detection method	sample size		Case group		Control group		HWE	NOS
				Case group	control group	GA+AA	GG	GA+AA	GG		
Abdallah A 2016	Saudi Arabia	case group (19.2 + 5.2) years old, control group (20.6 + 4.5) years old	PCR	118	200	99	19	169	31	coincidence	9
Settin A 2007	Egypt	5∼18 years old	PCR	50	98	38	12	93	5	coincidence	8
Rehman S 2013	Pakistan	case group (30 + 14.5) years old, control group (45 + 12.7) years old	PCR	150	204	101	49	140	64	coincidence	9

### Results of Meta-analysis

**Figure 2:**
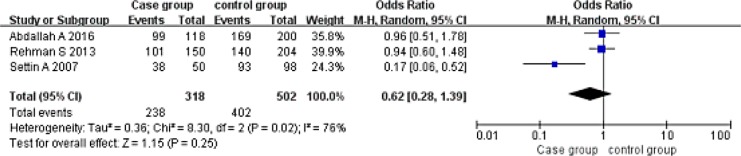
Meta-analysis of the correlation between the single nucleotide polymorphism of the IL-10-1082G/A site and rheumatic heart disease (AA+AG vs GG)

Dominant model (AA+AG vs GG). A total of 318 cases and 502 controls were included. With the genotype AA+AG as the exposure factor, the genotype GG was the non-exposure factor. Fixed effect model meta-analysis showed that I^2^ = 76%, *P* = 0.02, there was a large heterogeneity, so we use a random effects model. Random effects model meta-analysis showed that there was no association between the polymorphism of the gene and RHD [OR = 0.62, 95% CI (0.28, 1.39), *P* = 0.25]. As shown in Figure 2.

**Figure 3:**
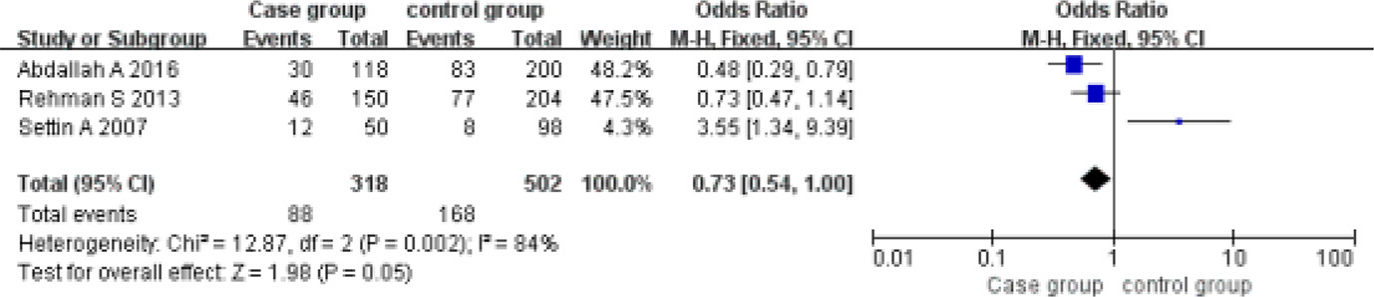
Meta-analysis of the correlation between the single nucleotide polymorphism of the IL-10-1082G/A site and rheumatic heart disease (AA vs AG+GG)

Implicit model (AA vs AG+GG). A total of 318 cases and 502 controls were included. With the genotype AA as the exposure factor, the genotype AG+GG was the non-exposure factor. Fixed effect model meta-analysis showed that I^2^ = 84%, *P* = 0.002. There is a large heterogeneity, so we use a random effects model. Random effects model meta-analysis showed that there was no association between the polymorphism of the gene and RHD [OR = 0.73, 95% CI (0.54, 1.00), *P* = 0.05]. As shown in Figure 3.

**Figure 4:**
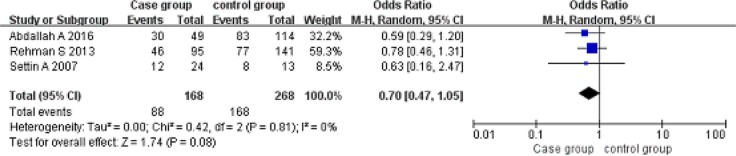
Meta-analysis of the correlation between the single nucleotide polymorphism of the IL-10-1082G/A site and rheumatic heart disease (AA vs GG)

Codominant model (AA vs GG). A total of 168 cancer patients and 268 controls were included. With the genotype AA as the exposure factor, the genotype GG was the non-exposure factor. Fixed effect model meta-analysis showed that I^2^ = 0%, *P* = 0.81, the heterogeneity was small, so we use a fixed effect model. Fixed effect model meta-analysis results showed that there was no association between the polymorphism of the gene and RHD [OR = 0.70, 95% CI (0.47, 1.05), *P* = 0.08]. As shown in Figure 4.

**Figure 5:**
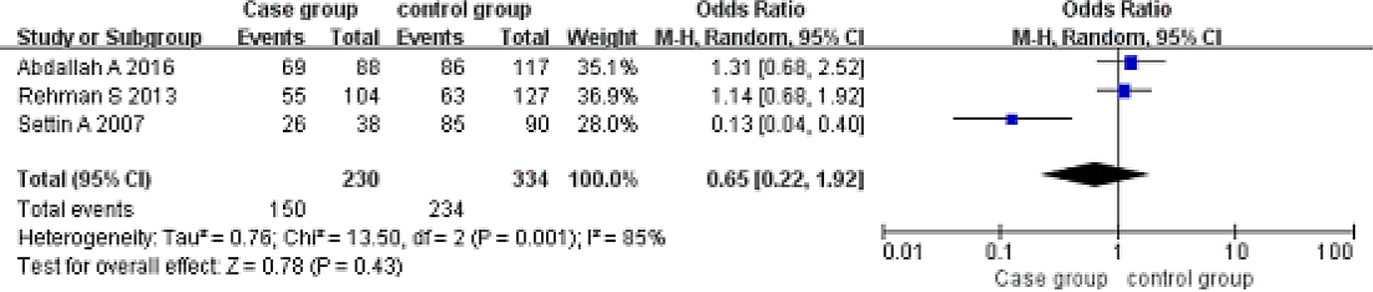
Meta-analysis of the correlation between the single nucleotide polymorphism of the IL-10-1082G/A site and rheumatic heart disease (AG vs GG)

Codominant model (AG vs GG). A total of 230 cancer patients and 334 controls were included. With the genotype AG as the exposure factor, the genotype GG was the non-exposure factor. Fixed effect model meta-analysis showed that I^2^ = 85%, *P* = 0.001, there is a large heterogeneity, so we use a random effects model. Random effects model meta-analysis showed that there was no association between the polymorphism of the gene and RHD [OR = 0.65, 95% CI (0.22, 1.92), *P* = 0.43]. As shown in Figure 5.

**Figure 6:**
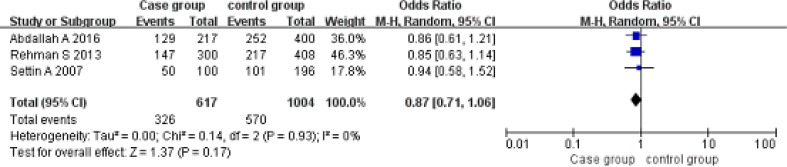
Meta-analysis of the correlation between the single nucleotide polymorphism of the IL-10-1082G/A site and rheumatic heart disease (A vs G)

Allele model (A vs G). A total of 308 patients and 502 controls were included. The allele G was the exposure factor, and the allele A was non-exposure factor. Fixed effect model meta-analysis showed that I^2^ = 0%, *P* = 0.93, the heterogeneity was small, so we use a fixed effect model. Fixed effect model meta-analysis results showed that there was no association between the polymorphism of the gene and RHD [OR = 0.87, 95% CI (0.71, 1.06), *P* = 0.17]. As shown in Figure 6.

## DISCUSSION

Interleukin 10 (IL-10) is a newly discovered cytokine in recent years, also known as cytokine synthesis inhibitory factor, with 178 amino acid residues in a single chain glycoprotein (N terminal 18 amino acid signal peptide sequence) [31–35]. IL-10 molecular weight 35-40 kDa, usually as a two polymer, its gene is single copy gene. Human IL-10 gene is located on chromosome 1, limiting endonuclease map indicated that the gene contains at least three interval sequences, with other known cytokines gene nucleotide sequence of goods corresponding protein sequence comparison of IL-10 and these cytokines no obvious sequence homology. IL-10 is mainly produced by Th2 cells, but Tho cells, Th cells, CD8+T cells, activated B cells, monocytes-macrophages, Kupffer cells, liver cells, keratinocytes can also produce.

Interleukin-10 (IL-10) is an important inflammatory factor in the body [36–39]. It has the function of anti-vascular growth and immune suppression. It also has the dual regulation effect of inhibiting tumor growth and promoting tumor growth. In recent years, more and more researches about the relationship between IL-10 polymorphism and disease have been studied. Among them, the correlation between IL-10-1082G/A polymorphism and rheumatic heart disease is one of the most important parts of the study [40–42]. Related research [43, 44] pointed out that the increase of IL-10 expression in the serum of patients with rheumatic heart disease is closely related to the occurrence and progression of the disease, and with the increase of the level of IL-10 expression, the worse the prognosis, the higher the mortality rate. Abdallah [30] study showed that the effect of IL-10 on the role of 1082G/A in patients with rheumatic heart disease is related to the single nucleotide polymorphisms (SNPs) in the gene promoter region. GG+GA gene polymorphism significantly increases the incidence of rheumatic heart disease in the population, AA gene polymorphism significantly reduces the incidence of rheumatic heart disease in the population, but the conclusion is still not uniform. Therefore, this study collect related case - control study and carries on the meta-analysis, further clarify the association between IL-10-1082G/A polymorphism and rheumatic heart disease, in order to provide a theoretical basis for the clear natural immunity and the pathogenesis of rheumatic heart disease.

The study included 3 case-control studies, a total of 318 patients with rheumatic heart disease and 502 control subjects. Our results showed that the IL-10-1082G/A polymorphism in the Implicit model (AA vs AG+GG) has a certain correlation with the risk of rheumatic heart disease, but dominant model (AA+AG vs GG), codominant model (AA vs GG), codominant model (AG vs GG) and Allele model (A vs G) have no associated with the risk of rheumatic heart disease. In this study, we found that there is a certain heterogeneity among the studies, which may including: ① The cases and controls involved in the study were derived from different regions, ethnic, and the Inclusion criteria among case group and control group are not agreement; ② The article Included in the study were published in the literature, not included the gray literature; ③ Different types of disease diagnosis methods from different countries or regions are different, which may bring about a certain heterogeneity.

The limitations of this study including: ① the incidence of rheumatic heart disease is related to many factors. But in this study, the correlation between one gene polymorphism and rheumatic heart disease was studied, and the interaction of gene and gene environment was analyzed, and the interaction of gene and gene environment was analyzed. ② Because of the small number of the included studies, the study was not further divided into subgroups according to the type of disease and race. If there are more studies in the future, we will further study the correlation between different types of disease and race. ③ The Meta analysis was included in the study population for the African population, and did not incorporate studies from Europe, Asia, and North America. So the conclusions of this study may not be applicable to people in Europe, Asia and North America.

In conclusion, this study found that the polymorphism of IL-10-1082G/A gene may be related to the risk of rheumatic heart disease. Due to the quantity and quality of the research, the conclusions of this study need to be carried out to verify the quality of the large sample.
